# Data on full wave-field measurement of transient guided resonance pulses propagating in a composite structure

**DOI:** 10.1016/j.dib.2019.104481

**Published:** 2019-09-05

**Authors:** Christophe Droz, Olivier Bareille, Mohamed Ichchou

**Affiliations:** Vibroacoustic and Complex Media Research Group, LTDS - CNRS UMR 5513, Ecole Centrale de Lyon, France

**Keywords:** Guided resonance, Cut-on, Wave mode, Actuation, Full wave-field measurement

## Abstract

Guided resonances (GR) are high-order, unidirectional waves able to propagate over a long range in finite cross-sections’ waveguides. Near cut-on GR exhibit a variety of scattering behaviors going from zero group velocity to quasi non-dispersive propagation.

This data article presents the surface velocities measured during the propagation of near cut-on high-order guided resonances in a 3-m-long sandwich panel. Full-field velocimetry is used to measure and visualize the transient propagation of 8-to-10 cycles wave pulses at 2500, 3000 and 4000 Hz. The waves are generated as transient pulses using waveform appropriation by an array of piezoelectric transducers at different bandwidths below and above the cut-on frequency. The data provided in this article includes the complete time-dependent wave-field measured during the propagation of the GR pulses.

This data will help testing and validating wave-based model updating or time-domain signal processing/filtering methodologies. One of the main contributions is to give researchers and industrial an insight on near cut-on GR scattering process occurring in the low acoustic (audible) frequency range.

**Table:** Specifications Table

Subject area	*Physics, Engineering*
More specific subject area	*Vibroacoustics, Wave propagation, NDE/SHM, Full-field velocimetry*
Type of data	Figures, 3D *.mat files, MATLAB Scripts, videos
How data was acquired	*Laser Doppler Velocimeter (PSV-400, Polytec scanning head and acquisition hardware)*
Data format	*Raw and Processed*
Experimental factors	*Capturing the full-field surface velocities during the propagation of transient guided resonances in a composite panel*
Experimental features	*Guided resonances are generated using a network of 14 PZT transducers in the low-frequency/audible range (2.5 – 4* *kHz). The panel is a 3 m-long carbon-reinforced sandwich structure with free boundary conditions. Transient wave pulses are measured on the entire surface of the panel with high spatial and temporal resolution.*
Data source location	*Data was obtained in the Vibration and Acoustics test facility at Ecole Centrale de Lyon, Ecully, France.*
Data accessibility	*Video data is available as supplementary files on the online version of this article. All data including the experimental datasets are available at: Droz, Christophe; Bareille, Olivier; Ichchou, Mohamed (2019), “Experimental dataset on guided resonance propagation”, Mendeley Data, v1*https://doi.org/10.17632/pbv5cmbwg9.1
Related research article	*Droz, C., Bareille, O., and Ichchou, M. N. (2019). Generation of long-range, near-cut-on guided resonances in composite panels. Journal of Applied Physics, 125(17), 175109.*

**Table:** 

**Value of the Data** - Complex propagating modes are widely studied in metamaterials and for SHM/NDE applications. This work provides the first open database describing near cut-on unidirectional guided resonances in a large composite waveguide. - This dataset gives insights on near cut-on scattering phenomena of complex guided resonances within the low acoustic bandwidth. - This article can be useful for researchers and industrials to understand the generation process of guided resonances using embedded PZT transducers. - This database will be used to test and validate wave-based models or time-domain signal processing/filtering techniques.

## 1 Data

Three pulses are generated and measured, respectively centered at: 2500 Hz (f1), 3000 Hz (f2) and 4000 Hz (f3). For each pulse, acquisition is made on 3484 measurement points, which are used to build a regular surface mesh of 30552 points (402 × 76) using spline interpolation. The database (see Ref. [2]) consists in 6 MATLAB arrays, used to create the figures and videos shown in the following. The general results, interpretations of the measurements and simulations are fully described in the research article [1].

1 “**time.mat**” (4096 samples) are the time samples from 0 to 20 ms with a 4.88 μs time step.
2 “**Xpos.mat**” (402 x-coordinates) are the positions of the data points along the propagation direction (x).
3 “**Ypos.mat**” (76 y-coordinates) are the position of the data points along the waveguide's width (y).
4 “**Scan1.mat**” (76 × 402 x 4096 double array) are the corresponding measured velocities for the pulse at f1: 2500 Hz.
5 “**Scan2.mat**” (76 × 402 x 4096 double array) are the corresponding measured velocities for the pulse at f2: 3000 Hz.
6 “**Scan3.mat**” (76 × 402 x 4096 double array) are the corresponding measured velocities for the pulse at f3: 4000 Hz.
7 “**SourceSignals.mat**” (4096 double arrays) are the input voltages provided by the amplifier for each pulse frequency (2500 Hz, 3000 Hz and 4000 Hz).
8 “**rawScans.mat**” contains the above data files without surface processing. Each point location corresponds to a laser acquisition point.

In addition, videos are provided for each bandwidth to help visualize the pulse scattering and conversion effects:

1 “**video1.avi**” displays the time-animated pulse propagation at f1: 2500 Hz.
2 “**video2.avi**” displays the time-animated pulse propagation at f2: 3000 Hz.
3 “**video3.avi**” displays the time-animated pulse propagation at f3: 4000 Hz.

**Fig. 1:**
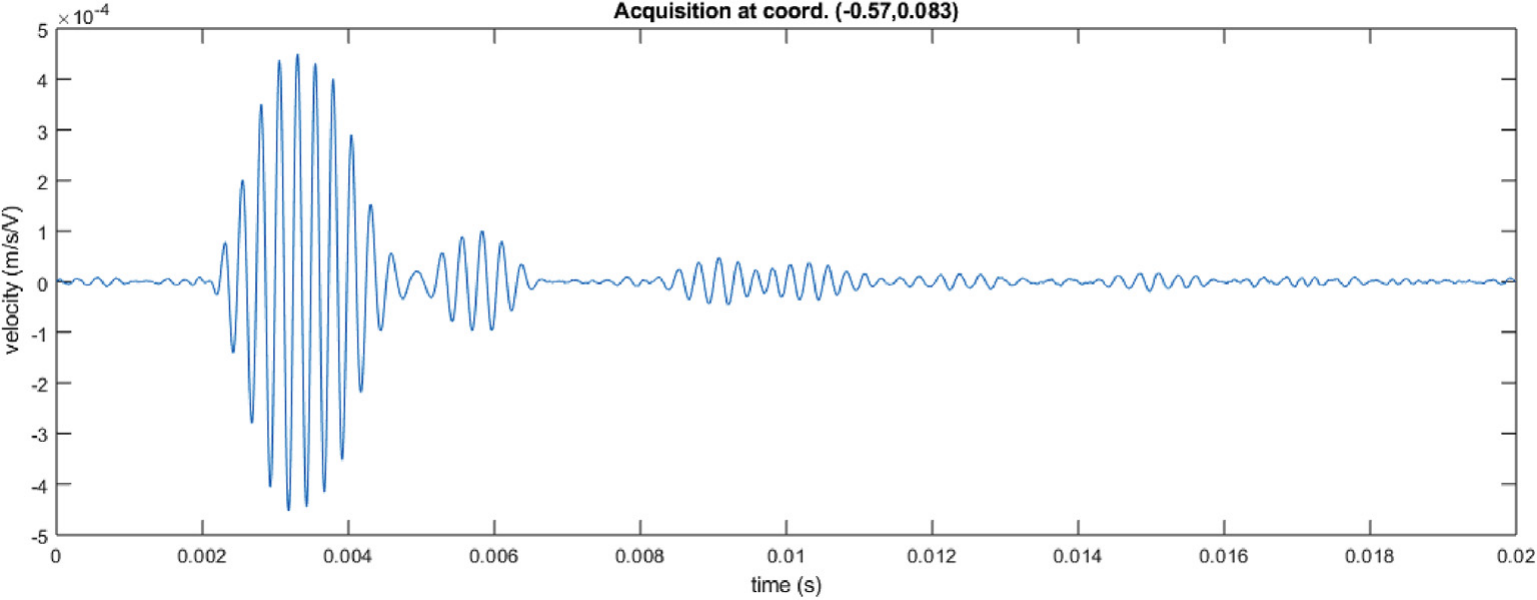
Time signal for Scan 3 (4000 Hz) at location index (indX = 100, indY = 36).

The following script 1 can be used for extracting time signals at location index (Xpos(i), Ypos(j)). The velocity signal captured at location indexes (100,36) is shown in Fig. 1. Location indexes can be arbitrarily chosen on the 402 × 76 grid. One can notice on Fig. 1 the incident wave pulse arrival at 2 ms, while successive reflections can be observed up to 18 ms. The presented time signals can easily be processed to determine the time-of-flight of each wave pulse, while wavelet analysis can be used to determine the dispersion properties of the sandwich waveguide.

**Figure:**
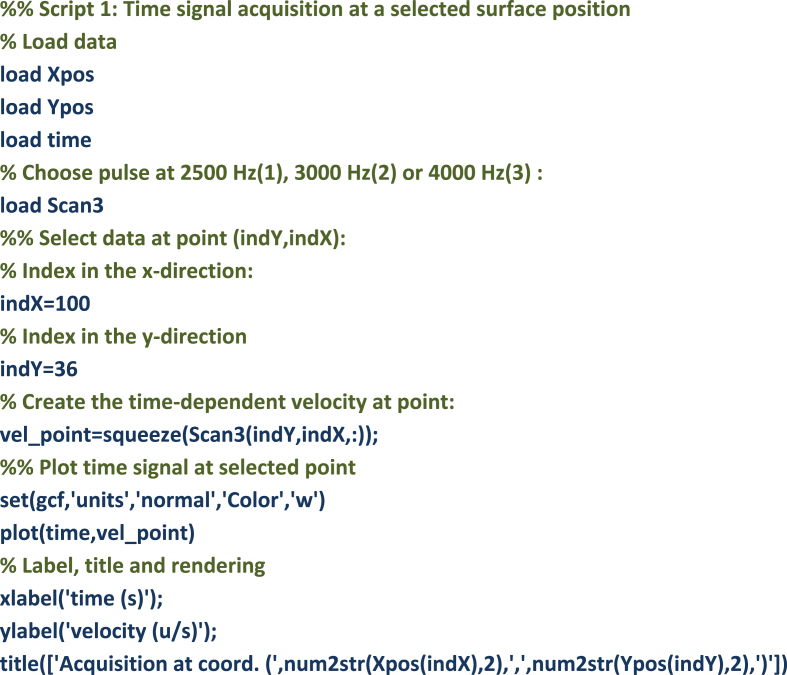

**Fig. 2:**
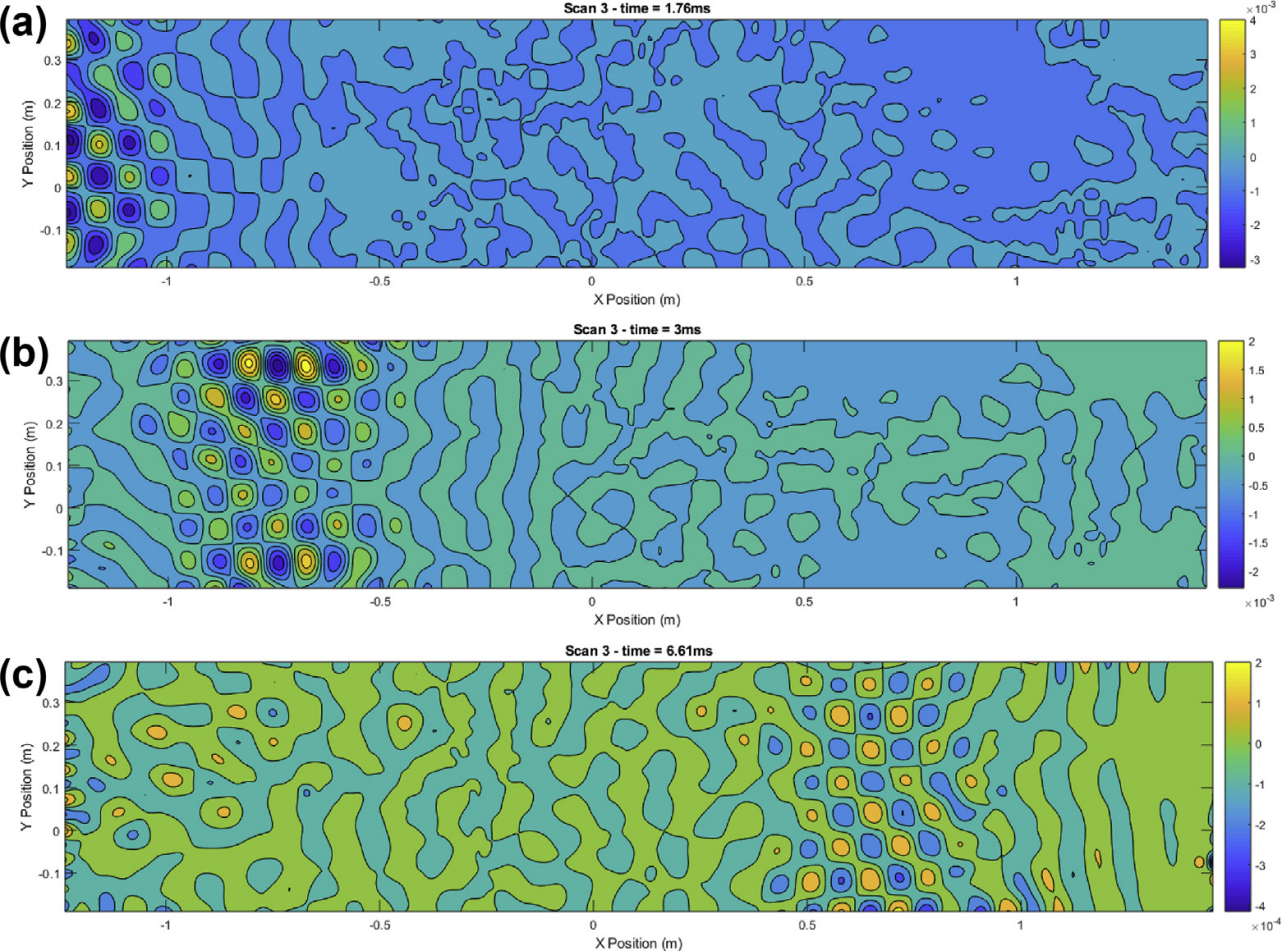
Time frames for pulse propagation at 4000 Hz. Time frames: (1.76 ms (a), 3 ms (b), 6.61 ms (c)).

**Fig. 3:**
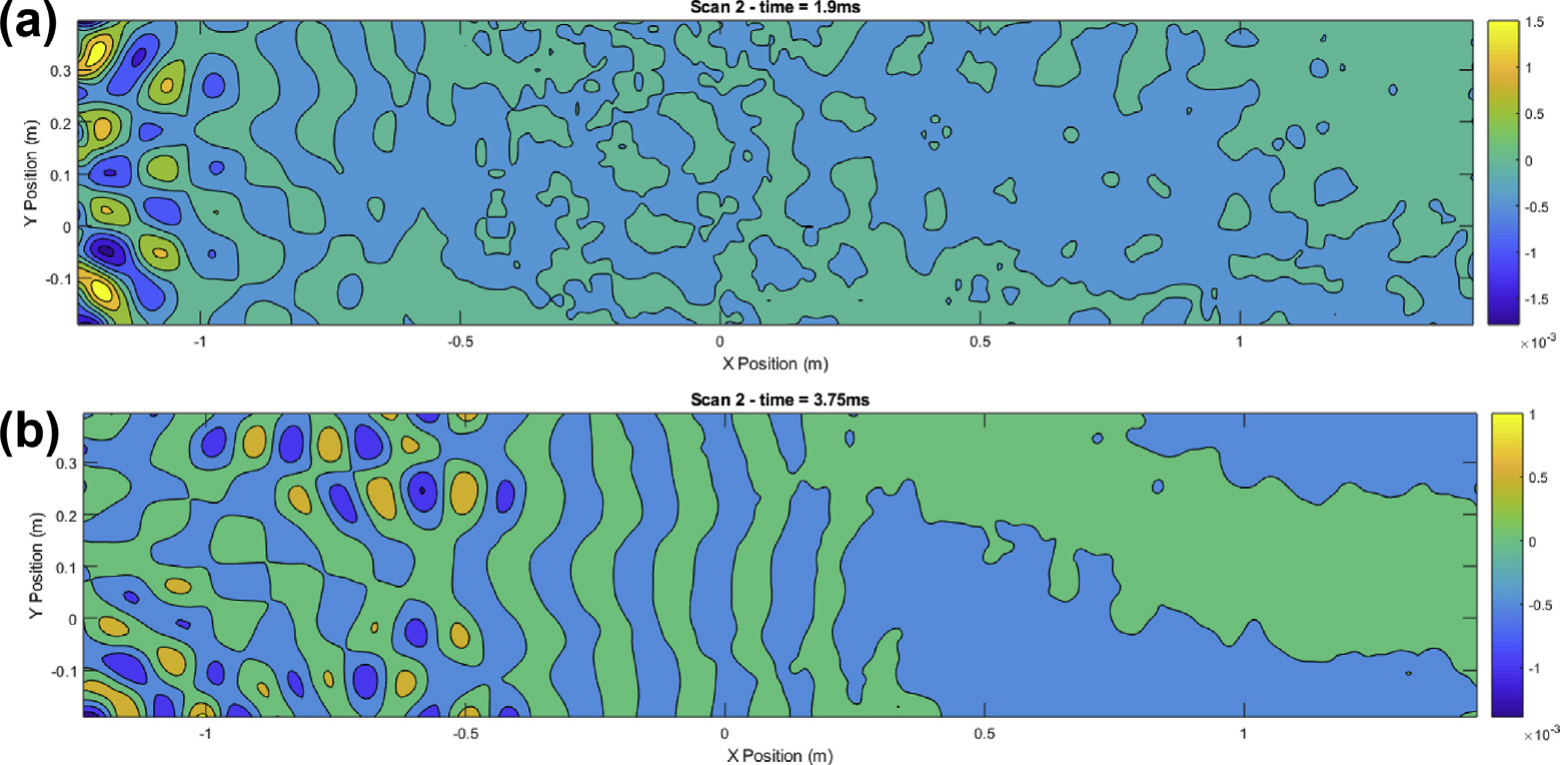
Time frames for pulse propagation at 3000 Hz. Time frames: (1.9 ms (a), 3.75 ms (b)).

**Fig. 4:**
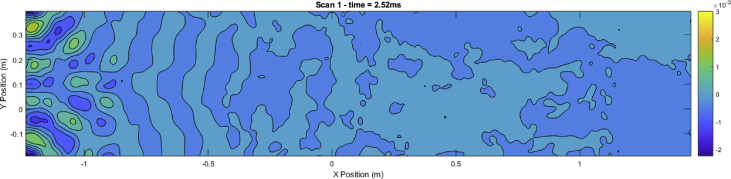
Time frame for pulse propagation at 2500 Hz. Time frame: 2.52 ms.

Script 2 is used to visualize the surface velocity wave-field at a selected time sample ‘indT’. Resulting plots are shown in Fig. 2, Fig. 3, Fig. 4. These results can be seen as the most valuable material of the present data article, as they provide a detailed description of the surface displacements of the sandwich structure at each of the 4096 time samples. The propagation of the wave pulses at 2500, 3000 and 4000 Hz can be observed, as well as the conversion process of the guided resonances during their propagation. It is emphasized that the guided resonances are actuated near their cut-on bandwidth, which results in the following observations: at 2500 Hz, the piezoelectric actuator is able to achieve a correct waveform appropriation but no propagation is observed beyond the actuated region. At 3000 Hz, slightly above the cut-on frequency, the selected guided resonance is actuated but fails to propagate due to a rapid conversion into low-order waves. At 4000 Hz, the wave exhibits a group velocity plateau, resulting in a low group velocity dispersion. The propagation of the wave pulse is therefore observed over the nearly 3 m.

**Figure:**
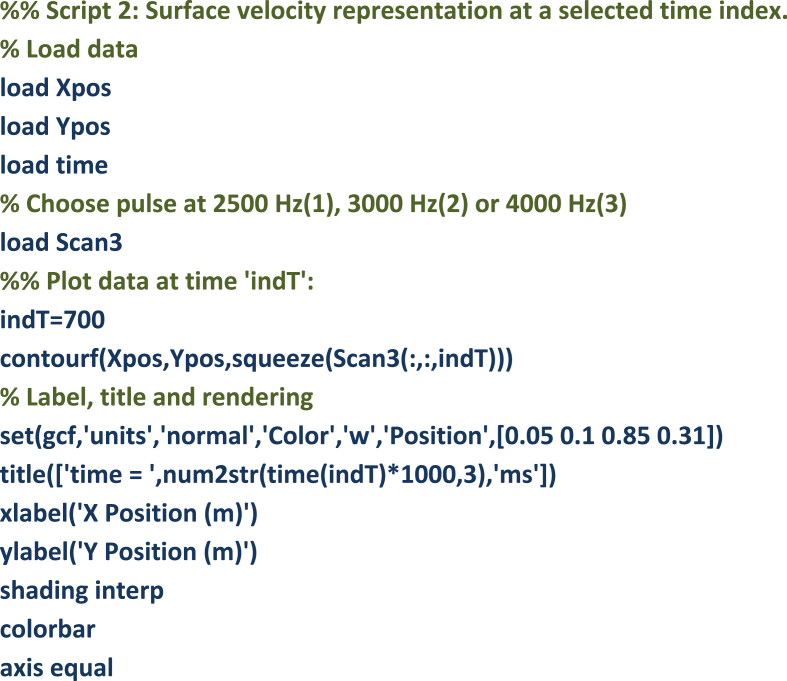

**Fig. 5:**
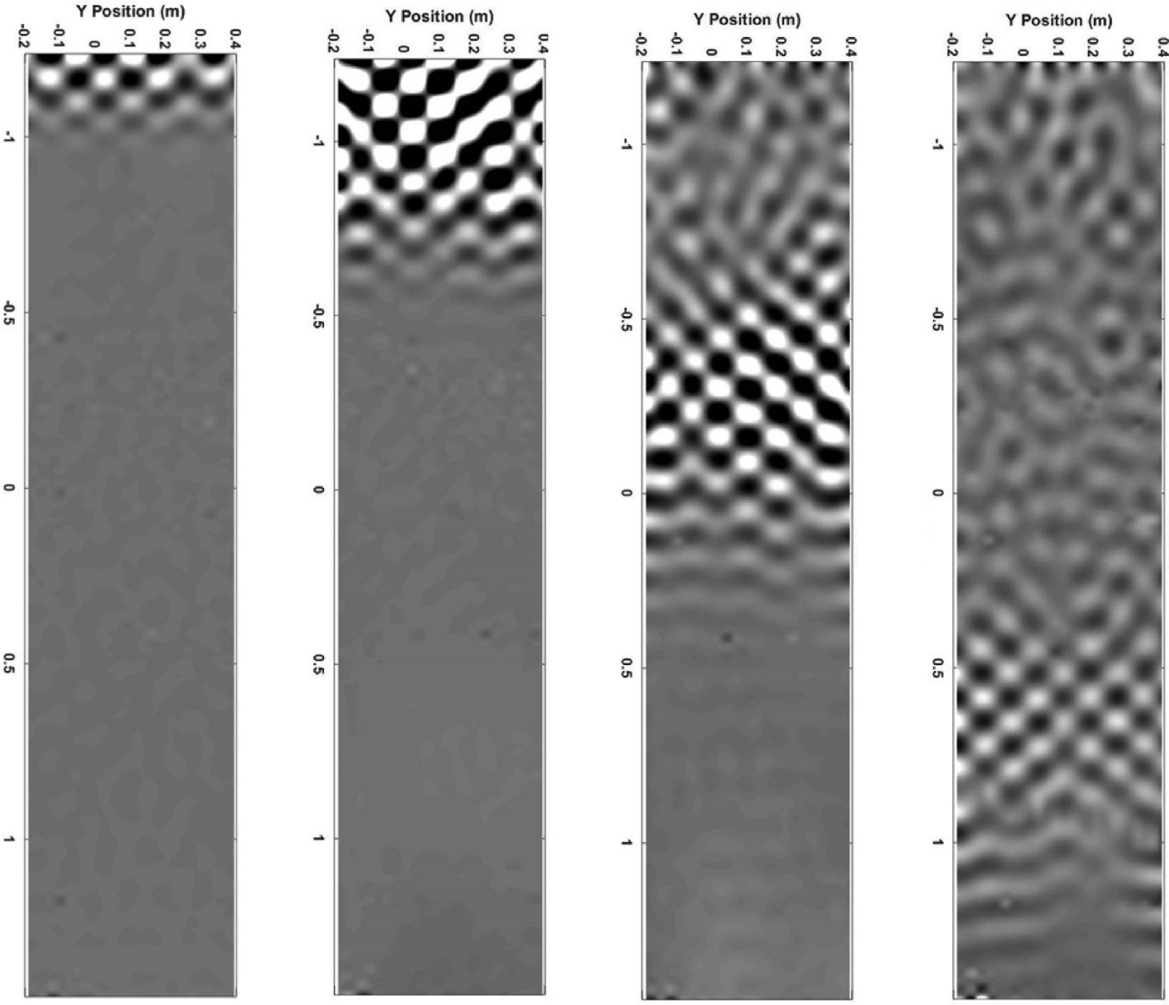
Frames of the video file “video3.avi” at selected times: 1.12, 2.12, 4.15, and 6.49 ms

The wave pulses are decaying along the propagation. To observe the conversion effects as a time-dependent phenomenon, the colormap can be changed to create animated plots. Time frames of the video at 4000 Hz are shown in Fig. 5. One video is available for each wave pulse frequency and the three videos together describe the overall collected dataset.

## 2 Experimental design, materials, and methods

### 2.1 Description of the composite panel

**Fig. 6:**
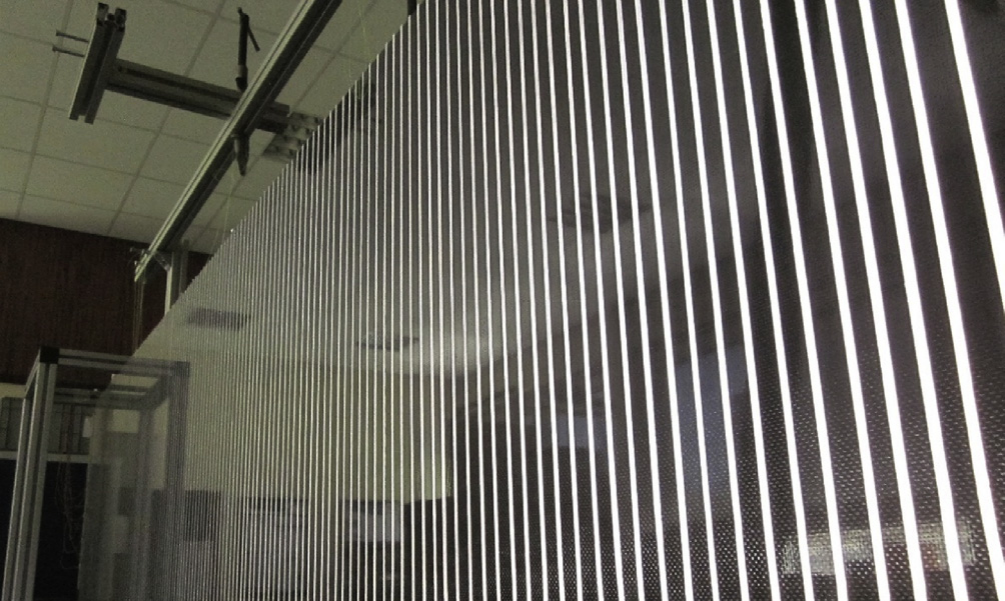
Reflexive tape covering the carbon-reinforced sandwich plate.

The composite waveguide is a sandwich plate of dimensions 600 × 2890 mm. The skins are 0.6 mm-thick carbon reinforced epoxy resin, while the core is a 10 mm-thick Nomex honeycomb structure (ECA, 3.2 mm cell size). The plate is suspended using strings in the width-vertical position. The panel is covered with thin reflexive strips to increase reflexivity with negligible effect on the panel's weight. The spacing between the strips is between 15 and 19 mm. The measurements are conducted on the entire width of the panel, and along the length starting at 57 mm from the right edge on the generator's side and ending at 33 mm from the left edge. A picture of the suspended tapered panel is shown in Fig. 6.

### 2.2 Generator

**Fig. 7:**
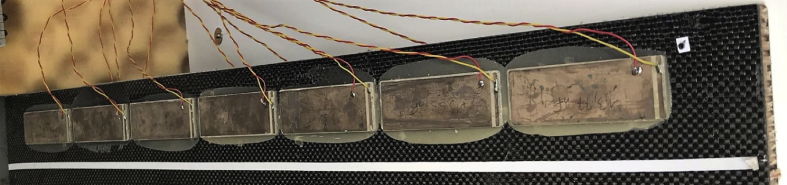
Patches used for waveform appropriation.

**Fig. 8:**
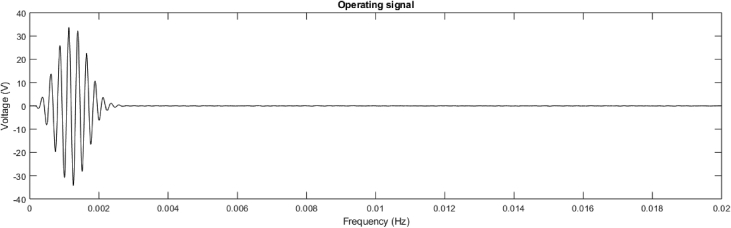
Input voltage signal for the pulse generation at 4000 Hz.

A total of 14 piezoelectric patches are distributed at a free edge of the plate and bonded on opposite lower and upper skins of with opposite polarities, and shown in Fig. 7. Details on the waveform appropriation technique are given in the associated research paper. The guided resonance generation technique consists in the application of loads associated with the shape of a propagating wave. The prototyping technique is further presented in Ref. [1]. The total mass of the panel with the embedded transducers is 4.55 kg. A mold is used to bond the patches using epoxy resin. The operating limits of the patches is 50 V in the considered bandwidth. The measured voltage at the edges of the patches is therefore set between 30 and 40 V for the three pulses at 2500, 3000 and 4000 Hz. The waveform of the input signal is given in Fig. 8 for the generated pulse at 4000 Hz.

### 2.3 Measurement set-up

**Fig. 9:**
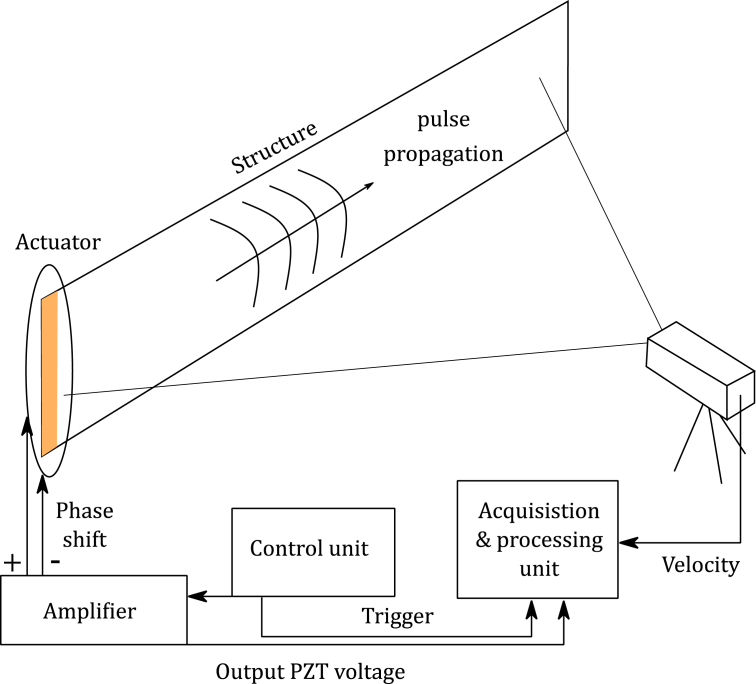
Description of the test set-up, wave generation and acquisition.

The test set-up is illustrated in Fig. 9. The pulses are generated using the actuation system located on the left side of the plate. Surface velocities are measured during the propagation of the guided resonances on the entire surface as described below. A PSV-I-400 MR Scanning Head (OFV-505) is placed in front of the panel to measure the normal velocities. The acquisition board is a PCI-4462 National Instruments with junction box PSV-E−401-H4. Signals are triggered and generated using the PSV control unit. Two amplifiers are used to control independently the two arrays of piezoelectric patches with a phase shift and measure the input voltage on each patch. The 3484 points are measured on a 0.6 × 2.8 m^2^ surface with a 4.88 μs time resolution. The sampling frequency is 204.8 kHz for a total acquisition time of 20 ms per sample. Each measurement is conducted 3 times and averaged. After each acquisition, the structure is put to rest for 1 second before next pulse generation. The total duration of the measurement was estimated to 21 hours (i.e. 7 hours for each transient acquisition), while the export of the raw data takes between 36 and 48 hours.

### 2.4 Post-processing & surface interpolation

The 3484 points measured on a 134 × 26 points grid are saved in a 3D-array with the mapping defined by vectors X and Y included in the file ‘rawScans.mat’. A refined mapped wavefield is created from these acquisition points using a spline interpolation. The regularly meshed velocity surface contains 402 × 76 points for a total of 30552 points.
